# Continued Stabilization of a Cementless 3D-Printed Total Knee Arthroplasty: Five-Year Results of a Randomized Controlled Trial Using Radiostereometric Analysis

**DOI:** 10.2106/JBJS.23.00221

**Published:** 2023-08-31

**Authors:** Thies J.N. van der Lelij, Perla J. Marang-van de Mheen, Bart L. Kaptein, Sören Toksvig-Larsen, Rob G.H.H. Nelissen

**Affiliations:** 1 Department of Orthopaedics, Leiden University Medical Center, Leiden, The Netherlands; 2 Medical Decision Making, Department of Biomedical Data Sciences, Leiden University Medical Center, Leiden, The Netherlands; 3 Department of Orthopaedics, Hässleholm Hospital, Hässleholm, Sweden; 4 Department of Clinical Sciences, Lund University, Lund, Sweden

## Abstract

**Background:**

Three-dimensional (3D) printing of highly porous orthopaedic implants aims to promote better osseointegration, thus preventing aseptic loosening. However, short-term radiostereometric analysis (RSA) after total knee arthroplasty (TKA) has shown higher initial migration of cementless 3D-printed tibial components compared with their cemented counterparts. Therefore, critical evaluation of longer-term tibial component migration is needed. We investigated migration of a cementless 3D-printed and a cemented tibial component with otherwise similar TKA design during 5 years of follow-up, particularly the progression in migration beyond 2 years postoperatively.

**Methods:**

Seventy-two patients were randomized to a cementless 3D-printed Triathlon Tritanium (Stryker) cruciate-retaining (CR) TKA or a cemented Triathlon CR (Stryker) TKA implant. Implant migration was evaluated with RSA at baseline and postoperatively at 3 months and at 1, 2, and 5 years. The maximum total point motion (MTPM) of the tibial component was compared between the groups at 5 years, and progression in migration was assessed between 2 and 5 years. Individual implants were classified as continuously migrating if the MTPM was ≥0.1 mm/year beyond 2 years postoperatively. Clinical scores were evaluated, and a linear mixed-effects model was used to analyze repeated measurements.

**Results:**

At 5 years, the mean MTPM was 0.66 mm (95% confidence interval [CI], 0.56 to 0.78 mm) for the cementless group and 0.53 mm (95% CI, 0.43 to 0.64 mm) for the cemented group (p = 0.09). Between 2 and 5 years, there was no progression in mean MTPM for the cementless group (0.02 mm; 95% CI, −0.06 to 0.10 mm) versus 0.07 mm (95% CI, 0.00 to 0.14) for the cemented group. One implant was continuously migrating in the cementless group, and 4 were continuously migrating in the cemented group. The clinical scores were comparable between the groups across the entire time of follow-up.

**Conclusions:**

No significant difference in mean migration was found at 5 years between the cementless and cemented TKA implants. Progression of tibial component migration was present beyond 2 years for the cemented implant, whereas the cementless implant remained stable after initial early migration.

**Level of evidence:**

Therapeutic Level I. See Instructions for Authors for a complete description of levels of evidence.

Although cemented fixation of an implant is predominantly used, the use of primary cementless total knee arthroplasty (TKA) continues to grow^1,2^. Observed loss of cement-bone interlock and debonding at the cement-implant interface contribute to the interest in cementless fixation^3,4^. With aseptic loosening as the leading cause of TKA revision, achieving long-lasting biological fixation of implants is important, especially in those who are ≤65 years of age as they may need durability of the implant for another 25 years^5,6^. The use of metallic 3D printing in orthopaedic surgery has become increasingly popular in the last decade; it enables the production of cementless implants with complex porous structures, which may contribute to enhanced bone-implant fixation^7-9^.

Excellent clinical outcomes at short-term and midterm follow-up have been described for cementless 3D-printed TKA implants with highly porous titanium, but it may take a longer time before problems with a particular device are shown in clinical outcomes^10-13^. Radiostereometric analysis (RSA) is a highly accurate method to detect implant migration, and it has been shown to predict future aseptic loosening^14,15^. RSA is well-suited for early detection of safety concerns, and it is the recommended technique for providing robust postmarketing surveillance^16,17^. Current evaluation of the 3D-printed Triathlon Tritanium TKA implant with the use of RSA remains limited to short-term follow-up, showing a higher initial migration compared with its cemented counterpart^18,19^. A recent case series documenting fatigue fractures of the 3D-printed tibial baseplate highlights possible safety concerns for this implant and underlines the importance of longer-term evaluation^20^.

This paper aims to compare tibial implant migration for up to 5 years postoperatively between the cementless 3D-printed TKA implant and its cemented counterpart, with a particular focus on the progression in migration beyond 2 years. We assessed whether implant migration was progressive over time or whether continuous stabilization was achieved after the initial “settling phase.” Our hypothesis was that both the cementless and cemented tibial components have no progression in migration beyond 2 years.

## Materials and Methods

### Design and Patients

This study was approved by the Regional Ethical Review Board in Lund, Sweden (entry no. 2015/8) and registered at ClinicalTrials.gov (NCT02578446). All of the patients gave informed consent prior to enrollment.

Patient selection and the surgical procedures that were used for this randomized RSA trial have been described previously^19^. In short, 72 patients were randomized to a cementless Triathlon Tritanium (Stryker) cruciate-retaining (CR) fixed-bearing TKA implant or a cemented Triathlon (Stryker) CR fixed-bearing TKA implant. The prostheses were identical in geometrical shape except for the 3D-printed porous structure and 4 pegs on the undersurface of the tibial baseplate of the cementless implant. SMARTSET GHV bone cement (DePuy Synthes) was used for the cemented group, leaving the tibial keel cementless in all cases. Eight spherical tantalum beads (diameter, 0.8 mm; RSA Biomedical) were inserted into the tibia, and 5 were inserted into the polyethylene of the tibial insert. Patients remained blinded to the treatment; the surgery was performed by a single experienced surgeon (S.T.-L.). Both groups received the same intraoperative treatment and postoperative rehabilitation, including immediate full weight-bearing on the day of surgery.

### Measurements

The baseline characteristics of the patients were collected, and RSA examinations were performed at baseline within 2 days after surgery as well as at 3 months and 1, 2, and 5 years postoperatively. RSA migration measurements were performed by 1 researcher (T.J.N.v.d.L.), blinded to clinical and patient-reported outcome measures. The Knee Society Score (KSS), the Knee injury and Osteoarthritis Outcome Score (KOOS), and the Forgotten Joint Score (FJS) were obtained at all of the follow-up times^21-23^. All scores range from 0 to 100, with higher scores indicating better outcomes.

### RSA

Radiographs were made with a biplanar technique at a 90° angle (Cage 10; RSA Biomedical) with the patient in the supine position. Analysis was performed with Model-based RSA software (version 4.2; RSAcore) and following RSA guidelines^24^. The precision of the local RSA setup was 0.1 mm for translations and 0.1° for rotations^19^. The largest set of consistent markers was used at each follow-up to assess migration of the tibial baseplate. The amount of translation of the marker with the greatest translation (i.e., the maximum total point motion [MTPM]) was used as the primary outcome measure^25^. Migration of the implant in patients who had a TKA in the left knee was transformed to match the data of those who had the TKA in the right knee. A mean error of rigid body fitting of ≤0.35 mm and a condition number of ≤120 were set as cutoff points^25^. Individual implants were considered to be “continuously migrating” if the MTPM was ≥0.3 mm (i.e., ≥0.1 mm/year) between 2 and 5 years postoperatively. Implants with ≥0.2 mm of micromotion in the second postoperative year but subsequent micromotion of <0.3 mm between 2 and 5 years were considered “stabilized.”^14,26,27^

### Statistical Analysis

As described previously, 23 patients were needed in each group to detect a difference between groups beyond the 0.13 mm measurement error of the MTPM with a power of 80% and an alpha of 0.05^19^. To account for possible dropouts and inadequate radiographs, 36 patients were randomized to each group.

The MTPM was compared between the TKA groups using a linear mixed-effects model (LMM), which effectively deals with missing values during follow-up or when patients withdraw from the study (e.g., due to revision); it also takes within-subject correlation into account. The model consisted of a group variable (cementless or cemented TKA implants), a time variable, and an interaction term between time and group. A random-intercept term was used, and any remaining variability was modeled with a heterogeneous autoregressive order-1 covariance structure. Given its non-normal distribution, the main outcome of MTPM was log-transformed and computed as log_10_(MTPM + 1). The values presented in this paper were then back-transformed to the original scale (mm). The mean MTPM at the 5-year follow-up was compared between the groups, and progression in MTPM beyond 2 years was assessed for each group. We evaluated the progression in migration by estimating the change in the MTPM from the LMM, using 3 months, 1 year, and 2 years as baselines. The delta method was used for approximating the standard error of the transformed mean differences. Descriptive RSA data of translations and rotations were presented to illustrate the direction of tibial component migration. Because a normal distribution could not be obtained through transformation for the clinical scores (KSS, KOOS, and FJS), a comparable generalized estimating equation (GEE) approach was used. Means were reported with 95% confidence intervals (CIs) or standard deviations (SDs). A p value of <0.05 was considered significant. Analysis was performed using SPSS (version 25.0; IBM) and R software (version 4.2.1; R Foundation for Statistical Computing).

### Cement Mantle Thickness

In a post hoc analysis, we explored the effect of cement mantle thickness on the migration of cemented tibial implants. Cement mantle thickness was evaluated by a single observer (T.J.N.v.d.L) at the first postoperative radiograph: 4 zones on the anteroposterior radiograph and 2 zones on the lateral radiographs were evaluated according to The Knee Society Roentgenographic Evaluation and Scoring System^28^. Measurements were performed at the tibial baseplate since the stem was not cemented. Because of random cement distribution that would have been affected by local bone architecture, cumulative measurements for the 6 zones were used^29,30^. The mean cumulative cement mantle thickness of the continuously migrating implants was compared with that of the non-continuously migrating cemented implants using an independent samples t test. An LMM, which included cement mantle thickness and time as covariates, was used to explore the association between cement mantle thickness and MTPM.

### Source of Funding

This investigator-initiated study was funded by Stryker, but Stryker employees had no part in the design, conduct, analysis, and interpretation of this study.

## Results

**Fig. 1:**
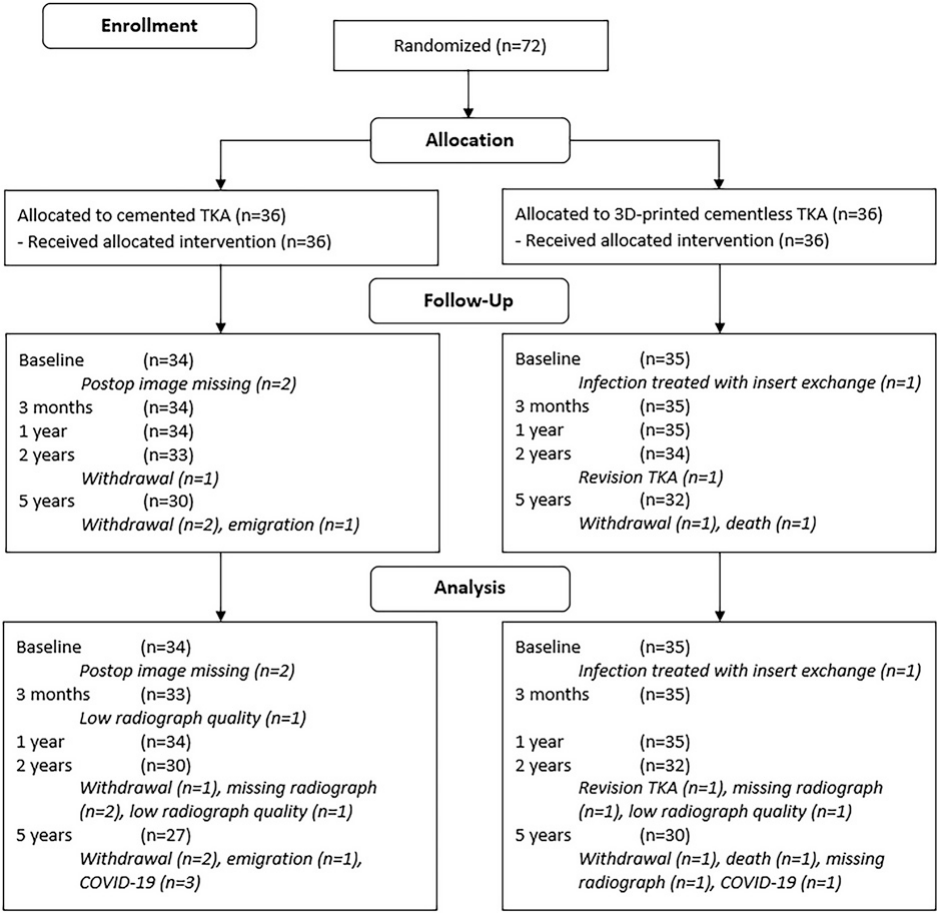
CONSORT (Consolidated Standards of Reporting Trials) flow diagram. TKA = total knee arthroplasty.

All 72 patients received the allocated intervention (Fig. 1). Postoperative RSA images of 2 patients in the cemented group were missing, and these patients were excluded from the analysis. In the cementless group, 1 insert was exchanged due to an infection at 3 weeks postoperatively. Because markers had been placed in the insert, this patient was excluded since RSA analysis could not be performed. Baseline characteristics of both groups are presented in Table I. In the cemented group, 3 patients withdrew from the study and 1 patient emigrated abroad during the 5-year follow-up. In the cementless group, 1 TKA was revised at 20 months postoperatively due to pain and migration of the tibial component, 1 patient withdrew from the study, and 1 patient died. Because of COVID-19, 3 patients in the cemented group and 1 patient in the cementless group were not able to visit the hospital for the 5-year follow-up; therefore, RSA examinations at the 5-year follow-up were performed on 27 and 30 patients in the cemented and cementless groups, respectively (Fig. 1).

**TABLE I:** Baseline Characteristics*

	Cemented (N = 34)	Cementless (N = 35)
Age *(yr)*	66 ± 6.3	65 ± 5.7
Male sex	18 (53)	18 (51)
BMI *(kg/m*^*2*^*)*	30 ± 3.1	28 ± 3.1
ASA classification		
I	4 (12)	13 (37)
II	26 (77)	21 (60)
III	4 (12)	1 (3)
Ahlbäck grade		
I	1 (3)	0 (0)
II	7 (21)	8 (23)
III	25 (74)	27 (77)
IV	1 (3)	0 (0)
Preoperative HKA angle		
Neutral†	1 (3)	4 (11)
Varus‡	30 (88)	23 (66)
Valgus§	3 (9)	8 (23)
Postoperative HKA angle		
Neutral†	23 (68)	20 (57)
Varus‡	6 (18)	9 (26)
Valgus§	5 (15)	6 (17)

* The values are given as the mean ± standard deviation or as the number with the percentage in parentheses. BMI = body mass index, ASA = American Society of Anesthesiologists, HKA = hip-knee-ankle.

† −3° to 3°.

‡ <−3°.

§ >3°.

### RSA Migration Measurements

**Fig. 2:**
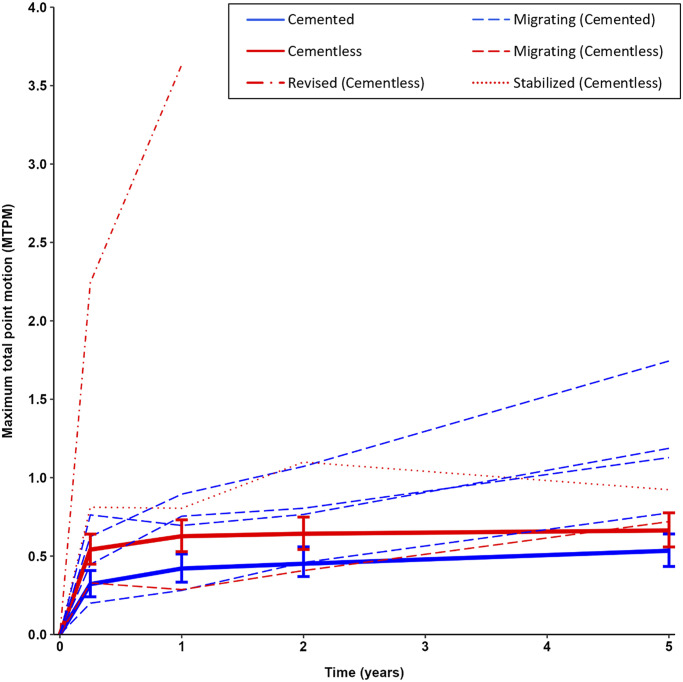
MTPM during the 5-year follow-up. The solid lines represent the mean MTPM of the groups, with 95% confidence intervals for all patients. Separate lines are presented for the individual revised, continuously migrating, and stabilized implants.

The mean MTPM of the cementless and cemented groups at the 5-year follow-up was 0.66 mm (95% CI, 0.56 to 0.78 mm) and 0.53 mm (95% CI, 0.43 to 0.64 mm), respectively (p = 0.09). Between 2 and 5 years, there was no progression in mean MTPM for the cementless group (0.02 mm; 95% CI, −0.06 to 0.10 mm) versus 0.07 mm (95% CI, 0.00 to 0.14 mm) for the cemented group (Table II). Similarly, taking 1 year as the baseline, the cementless components showed no progression in MTPM between 1 and 5 years (0.04 mm; 95% CI, −0.06 to 0.13 mm), whereas the cemented component did show progression (0.11 mm; 95% CI, 0.05 to 0.20 mm). Differences in MTPM between the cementless and cemented groups became smaller over time (Fig. 2). At 3 months and at 1, 2, and 5 years of follow-up, the difference in MTPM was 0.22 mm (95% CI, 0.09 to 0.34 mm), 0.21 mm (95% CI, 0.07 to 0.34 mm), 0.18 mm (95% CI, 0.04 to 0.32 mm), and 0.13 mm (95% CI, −0.02 to 0.28 mm), respectively. Translations along and rotations about each of the orthogonal axes are presented in the Appendix, showing a greater absolute initial subsidence of the cementless implant, although it remained stable beyond 2 years.

**TABLE II:** Progression in MTPM*

		Cemented	Cementless
Postoperative	3 mo	0.32 (0.24 to 0.41)	0.54 (0.45 to 0.64)
	1 yr	0.42 (0.33 to 0.51)	0.63 (0.53 to 0.73)
	2 yr	0.46 (0.37 to 0.56)	0.64 (0.54 to 0.75)
	5 yr	0.53 (0.43 to 0.64)	0.66 (0.56 to 0.78)
3 mo	1 yr	0.10 (0.04 to 0.16)	0.09 (0.02 to 0.15)
	2 yr	0.14 (0.06 to 0.22)	0.10 (0.01 to 0.19)
	5 yr	0.21 (0.11 to 0.31)	0.12 (0.01 to 0.23)
1 yr	2 yr	0.04 (−0.02 to 0.11)	0.02 (−0.05 to 0.09)
	5 yr	0.11 (0.05 to 0.20)	0.04 (−0.06 to 0.13)
2 yr	5 yr	0.07 (0.00 to 0.14)	0.02 (−0.06 to 0.10)

* The change in MTPM between the selected baseline and the specific follow-up moment was derived from the linear mixed-effects model and back-transformed to the original scale (mm).

**Fig. 3:**
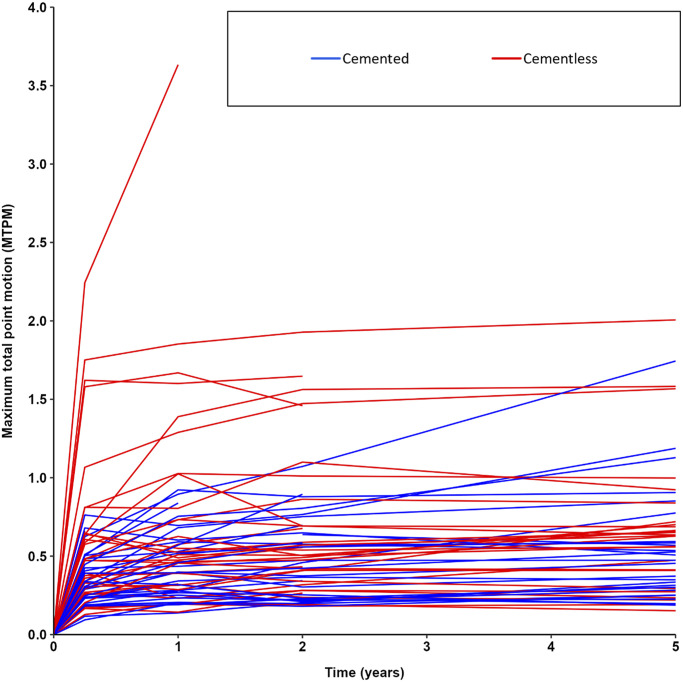
Spaghetti plot showing the individual implant-migration profiles.

**Fig. 4:**
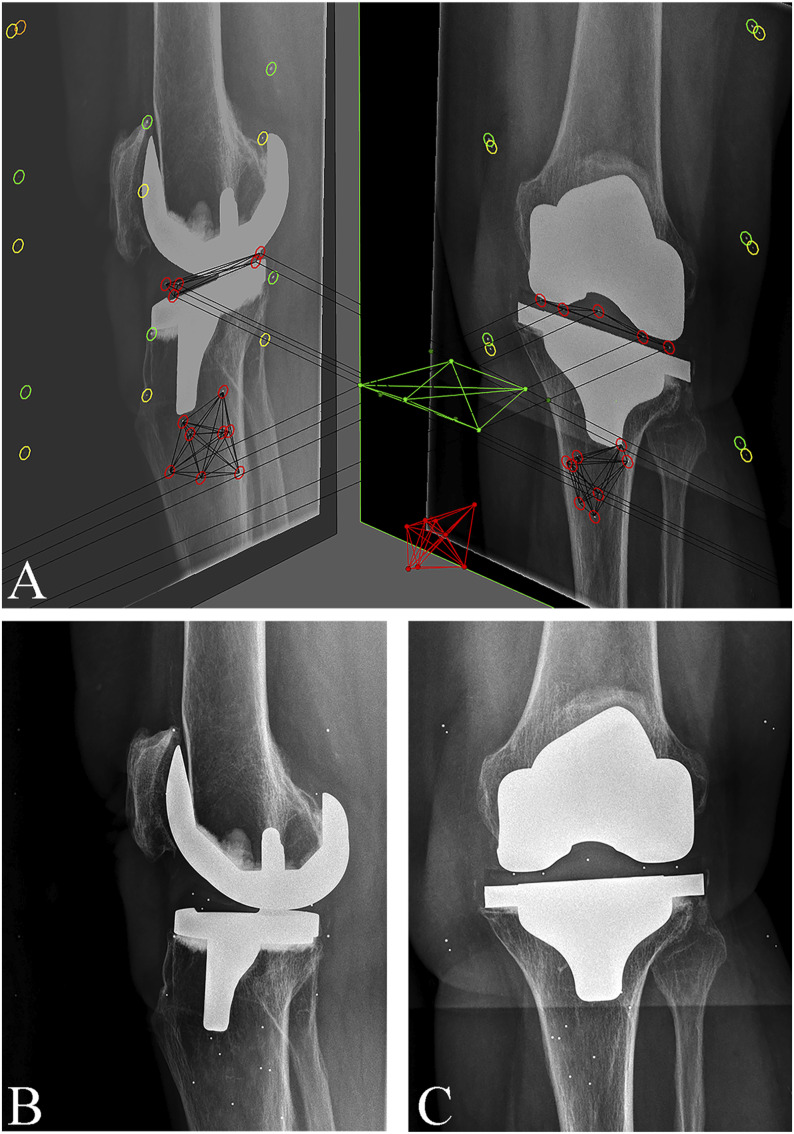
**Figs. 4-A, 4-B, and 4-C** RSA images of a cemented TKA implant. **Fig. 4-A** Biplanar (lateral and anteroposterior) views with markers inserted in the polyethylene insert and tibial bone. **Fig. 4-B** Lateral radiograph of the same implant, which was classified as continuously migrating. **Fig. 4-C** Anteroposterior radiograph of the same implant.

Multiple cementless implants showed high initial migration in the first 3 months but stabilized before the second postoperative year (Fig. 3). Nevertheless, these implants contributed to the (higher) overall mean migration of the cementless TKA group (Fig. 2). One cementless and 4 cemented components showed continuous migration beyond 2 years (Figs 2 and 4). One cementless component showing ≥0.2 mm of migration in the second postoperative year showed no further progression beyond 2 years and was therefore classified as stabilized. One patient (not shown in Fig. 2) with a cemented component showed ≥0.2 mm of migration in the second postoperative year but missed the 5-year follow-up visit because of COVID-19, and, therefore, the implant could not be classified as either stabilized or continuously migrating.

### Cement Mantle Thickness

Given the progression in mean MTPM for the cemented group beyond 2 years, we explored whether this could be explained by the immediate postoperative cement mantle thickness. The mean cement mantle thickness of the 4 continuously migrating implants (10.11 mm; SD, 4.1) and of the 30 non-migrating cemented implants (9.95 ± 3.5 mm) were comparable (p = 0.94). LMM analysis showed no association between cement mantle thickness and MTPM across the 5-year follow-up period (p = 0.86).

### Clinical Scores and Patient-Reported Outcome Measures

**Fig. 5:**
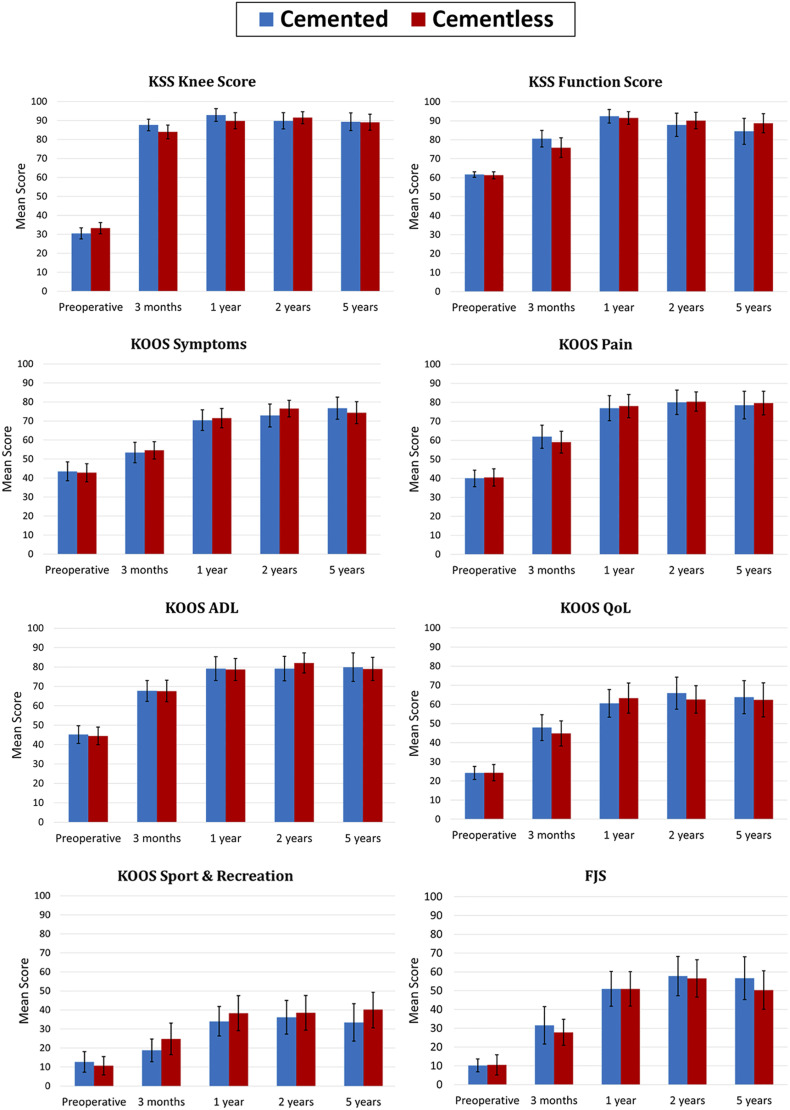
Mean clinical scores with 95% confidence intervals. KSS = Knee Society Score, KOOS = Knee injury and Osteoarthritis Outcome Score, ADL = activities of daily living, QoL = quality of life, and FJS = Forgotten Joint Score.

No significant differences between the groups were found during the 5-year follow-up for the KSS Knee score, the KSS Function score, the KOOS subscales, and the FJS (Fig. 5).

## Discussion

The present study showed no difference in mean MTPM between the cementless 3D-printed and cemented tibial implants at 5 years postoperatively, despite higher initial migration of the 3D-printed implant. While the cemented implant had less migration in the early postoperative period, it showed progression of migration beyond 1 year, whereas the cementless implant remained stable. Moreover, only 1 cementless implant was continuously migrating beyond 2 years versus 4 cemented implants. This was probably related to a strong implant-bone interlock in the cementless design and a less favorable cement-bone interlock in the cemented implant.

Previous studies have reported excellent clinical outcomes of the 3D-printed implant at short-term and midterm follow-up, which was confirmed by our results^10-13^. Although 2 years has generally been accepted as the benchmark for measuring migration, few data are available on this novel cementless 3D-printed implant, stressing the importance of evaluating migration over time^31^. To our knowledge, this study is the first to report migration results of the Triathlon Tritanium TKA implant beyond 2 years. In a cohort study with 2 years of follow-up, Sporer et al. analyzed the migration of the 3D-printed Triathlon Tritanium TKA implant in 29 patients with use of RSA; they found no significant progression in mean MTPM between 1 and 2 years^18^. Our study found that this pattern continued to 5 years of follow-up. RSA studies investigating cementless designs typically have shown higher early migration, in the first postoperative year (the settling phase), compared with cemented implants^19,32,33^. Cemented implants usually show little early migration since they rely on primary bone fixation through cement interdigitation^15^. However, cemented implants are susceptible to cement-related complications, including concerns regarding loosening caused by tension and shear as well as third-body wear from cement debris^5^. A previous study described an equivalent migration pattern for cemented and cementless tibial components between 1 and 2 years postoperatively^34^. RSA studies with longer follow-up are scarce and inconclusive regarding the migration of cemented implants. For example, Nilsson et al. found that cementless components stabilized after an initial period of early migration whereas cemented implants showed initially lower migration followed by progressive migration beyond 2 years^35^. However, other RSA studies have rarely shown continuous migration of cemented tibial implants beyond 2 years^27,36,37^.

Two RSA studies with at least 5 years of follow-up using the same cemented TKA design have been performed in the same hospital as the present study (Hässleholm Hospital)^26,32^. Consistent with our results, van Hamersveld et al. showed progression in migration of the cemented component, with a mean MTPM of 0.58 ± 0.35 mm at 2 years to 0.68 ± 0.50 mm at 5 years^32^. In contrast, Molt et al. reported a similar mean MTPM at 2 and 5 years (0.65 ± 0.66 and 0.66 ± 0.38 mm, respectively), but did not employ an LMM to deal with missing values and repeated measurements, which may have affected their results^26^. Both studies did not specifically report the progression in mean MTPM (and corresponding 95% CI) between 2 and 5 years. Studies focusing only on between-group comparisons may overlook significant changes in migration over time within 1 group.

Significant progression in MTPM does not directly imply a clinically relevant increase in the rate of aseptic loosening. Still, the migration of the cemented implant beyond 2 years was unexpected and warrants further research. Cement mantle thickness and proper penetration of cement into bone have been suggested to influence implant stability^38-41^. However, we found no influence of cement mantle thickness on tibial implant migration. The results of that post hoc analysis, however, should be regarded as exploratory. For a definitive answer regarding whether there is an association between cement mantle thickness and implant migration, a clinical study is needed that has sufficient power and includes analysis of inter- and intraobserver variability. Additionally, it is important to note that measurements of cement mantle thickness do not represent the quantity of fixation from the cement-bone interface^3^. Interestingly, all of the patients with a cemented implant that showed continuous migration were female. Female sex has been described as a risk factor for increased migration of cemented implants during the first 3 months^42^. Laende et al. found that larger tibial components were associated with increased migration for cemented implants in women^43^, but this was not observed in our study.

A strength of this study was the use of RSA for a highly accurate measurement of implant migration. Besides the comparison of 2 TKA designs, RSA allows for analysis of implant migration over time within 1 group. However, some limitations should be noted. The effect of the 3D-printed cementless design cannot be separated from that of the 4 additional pegs on the undersurface of the tibial plateau^19^. To specifically assess the effect of 3D printing on implant migration, a comparison is needed with a conventionally manufactured cementless TKA implant instead of a cemented TKA implant. Also, the study was single-blinded since it is impossible to blind clinicians and researchers given the difference in radiographic appearance of the 2 types of implants.

In conclusion, there was no progression in MTPM for the cementless 3D-printed tibial components between 2 and 5 years, whereas the cemented components showed progression in migration. The early postoperative migration of the cementless 3D-printed TKA components occurred mainly during the first 3 months and can probably be considered physiological as part of the implant settling phase.

## Appendix

Supporting material provided by the authors is posted with the online version of this article as a data supplement at jbjs.org (http://links.lww.com/JBJS/H672).

## Data Availability

A **data-sharing statement** is provided with the online version of the article (http://links.lww.com/JBJS/H673).
